# Supplementary material for: Polyunsaturated Phospholipids Increase Cell Resilience to Mechanical Constraints
Source: Cells. 2021 Apr 17;10(4):937. doi: 10.3390/cells10040937 (PMC8073313; doi:10.3390/cells10040937)

Supplementary figure titles and legends

Supplementary Figure 1: PC species distribution in the various organs.

Total lipids were extracted and phospholipid species were purified and analyzed by ESI-MS from samples corresponding to the indicated organs, as described in the "Materials and Methods" section. PC subspecies distribution in each case are displayed. The total carbon chain length (x) and number of carbon-carbon double bounds (y) of the main PC molecular species (x:y) are indicated. Values are means ± S.D. of four independent determinations from four individuals from both groups in each case.

Supplementary Figure 2: PI species distribution in the various organs.

Total lipids were extracted and phospholipid species were purified and analyzed by ESI-MS from samples corresponding to the indicated organs, as described in the "Materials and Methods" section. PI subspecies distribution in each case are displayed. The total carbon chain length (x) and number of carbon-carbon double bounds (y) of the main PC molecular species (x:y) are indicated. Values are means ± S.D. of four independent determinations from four individuals from both groups in each case.

Supplementary Figure 3: PE species distribution in the various organs.

Total lipids were extracted and phospholipid species were purified and analyzed by ESI-MS from samples corresponding to the indicated organs, as described in the "Materials and Methods" section. PE subspecies distribution in each case are displayed. The total carbon chain length (x) and number of carbon-carbon double bounds (y) of the main PC molecular species (x:y) are indicated. Values are means ± S.D. of four independent determinations from four individuals from both groups in each case.

Supplementary Figure 4: PS species distribution in the brain and the muscle.

Total lipids were extracted and phospholipid species were purified and analyzed by ESI-MS from samples corresponding to the indicated organs, as described in the "Materials and Methods" section. PS subspecies distribution in each case are displayed. The total carbon chain length (x) and number of carbon-carbon double bounds (y) of the main PC molecular species (x:y) are indicated. Values are means ± S.D. of four independent determinations from four individuals from both groups in each case.

Supplementary Figure 5: PC species distribution in C2C12 cells grown under various fatty acid supplementations.

Total lipids were extracted and phospholipid species were purified and analyzed by ESI-MS from samples corresponding to C2C12 cells grown under the indicated conditions, as described in the "Materials and Methods" section. PC subspecies distribution in each case are displayed. The total carbon chain length (x) and number of carbon-carbon double bounds (y) of the main PC molecular species (x:y) are indicated. Values are means ± S.D. of four independent determinations from four individuals from both groups in each case.

Supplementary Figure 6: PC Double-Bond (DB) index and DHA to AA ratios in C2C12 cells grown under various fatty acid supplementations.

The relative percentage of saturated (DB=0: no double bonds) versus monounsaturated (DB=1: one double bond), diunsaturated (DB=2: two double bonds) and polyunsaturated (DB > 2: > two double bonds) PhosphatidylCholine (PC) species was obtained from the PC subspecies distribution displayed

in Supplementary Fig. 5. The ratio of Arachidonic Acid (AA)- to Docosahexaenoic Acid (DHA)-containing PC subspecies in the various organs is also displayed.

Supplementary Movie 1

Dynamic examples of transmission (left images) and fluorescence at 515 nm (right images) images of FM1-43-labelled myoblasts cultivated either in DHA-supplemented (below) or not-supplemented (NT) (above) media and submitted to osmotic shock. Images were recorded every five seconds for 25 minutes.

Supplementary Movie 2

Dynamic examples of cell breakage following osmotic downshock: when the integrity of cell membrane is lost, FM1-43 rapidly fills the cytoplasm and generates an intense fluorescence at 543 nm. Representative transmission (left) and fluorescence (543 nm) series of images (right) of FM1-43-labelled. Images were recorded every five seconds for 25 minutes. White arrows designate two cell breakages.

Supplementary Fig. 1

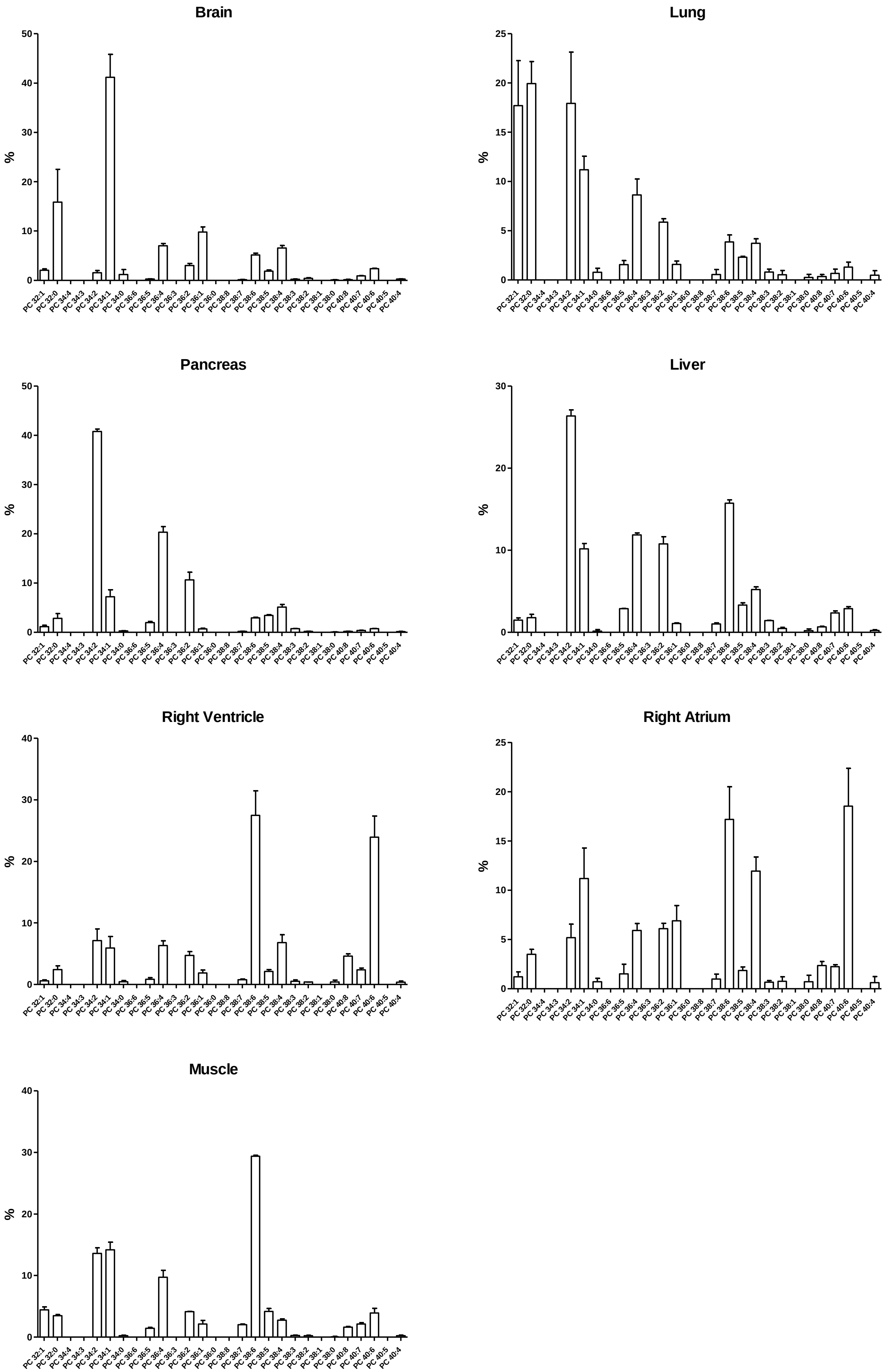

Supplementary Fig. 2

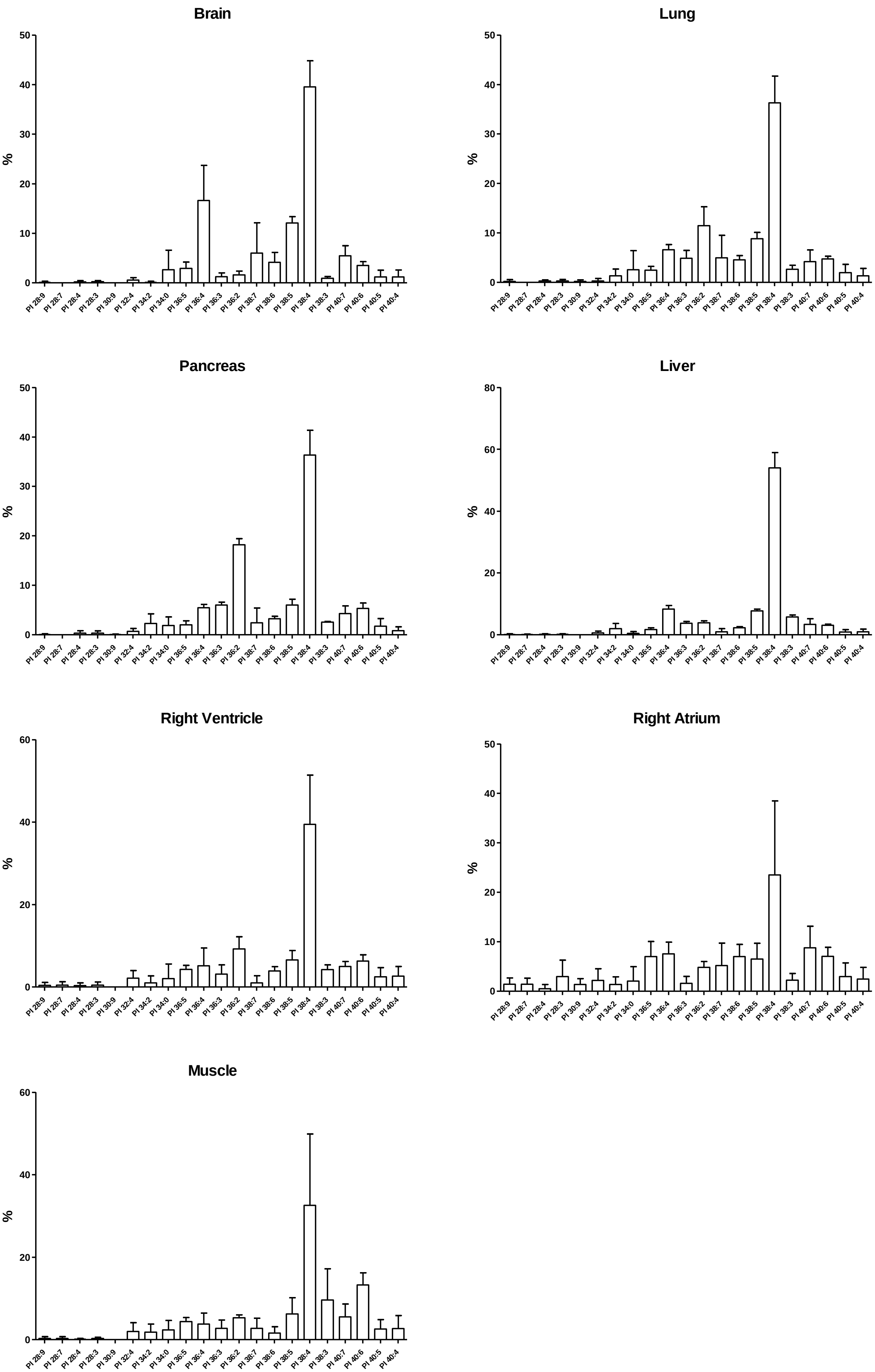

Supplementary Fig. 3

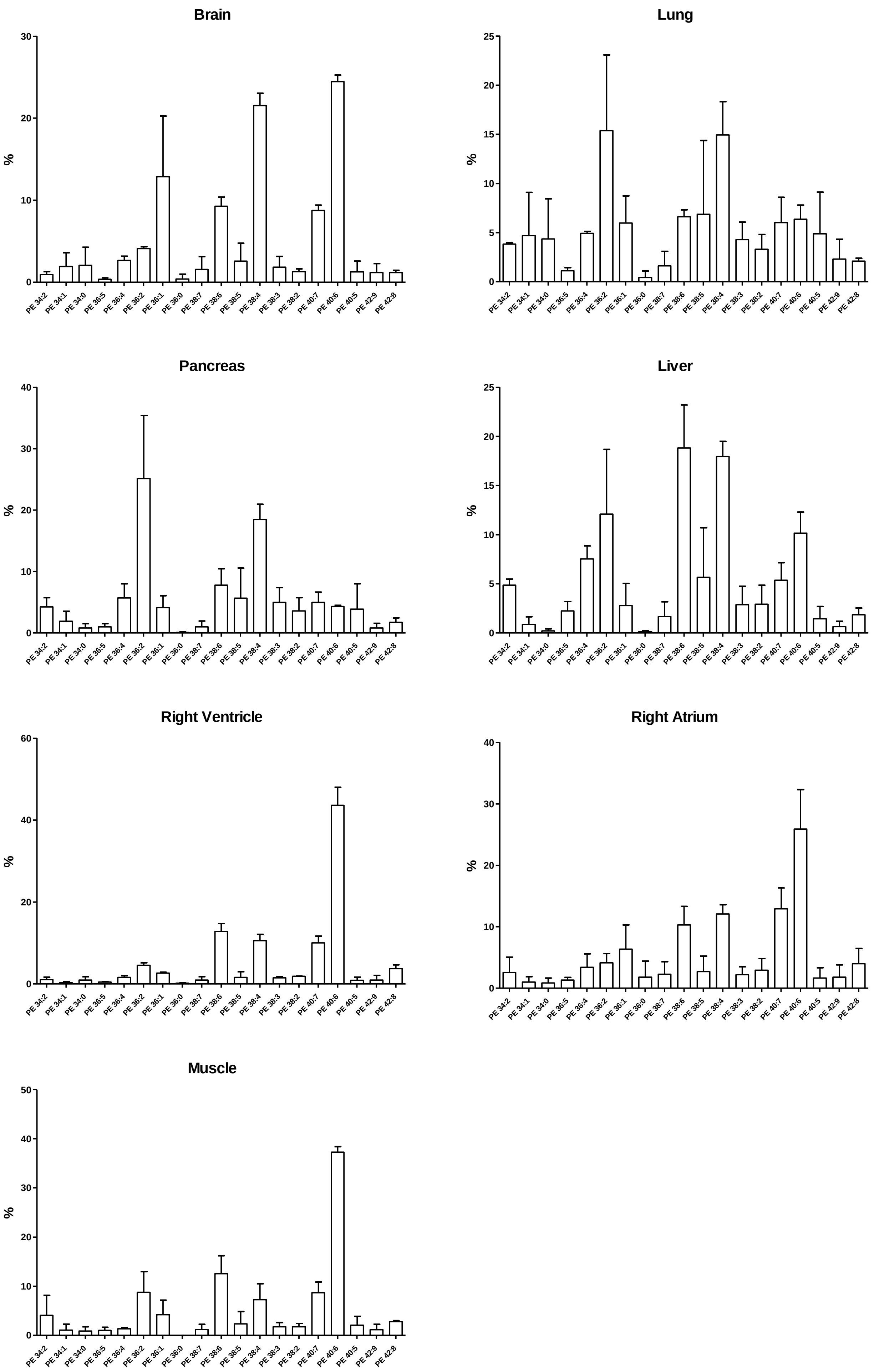

Supplementary Fig. 4

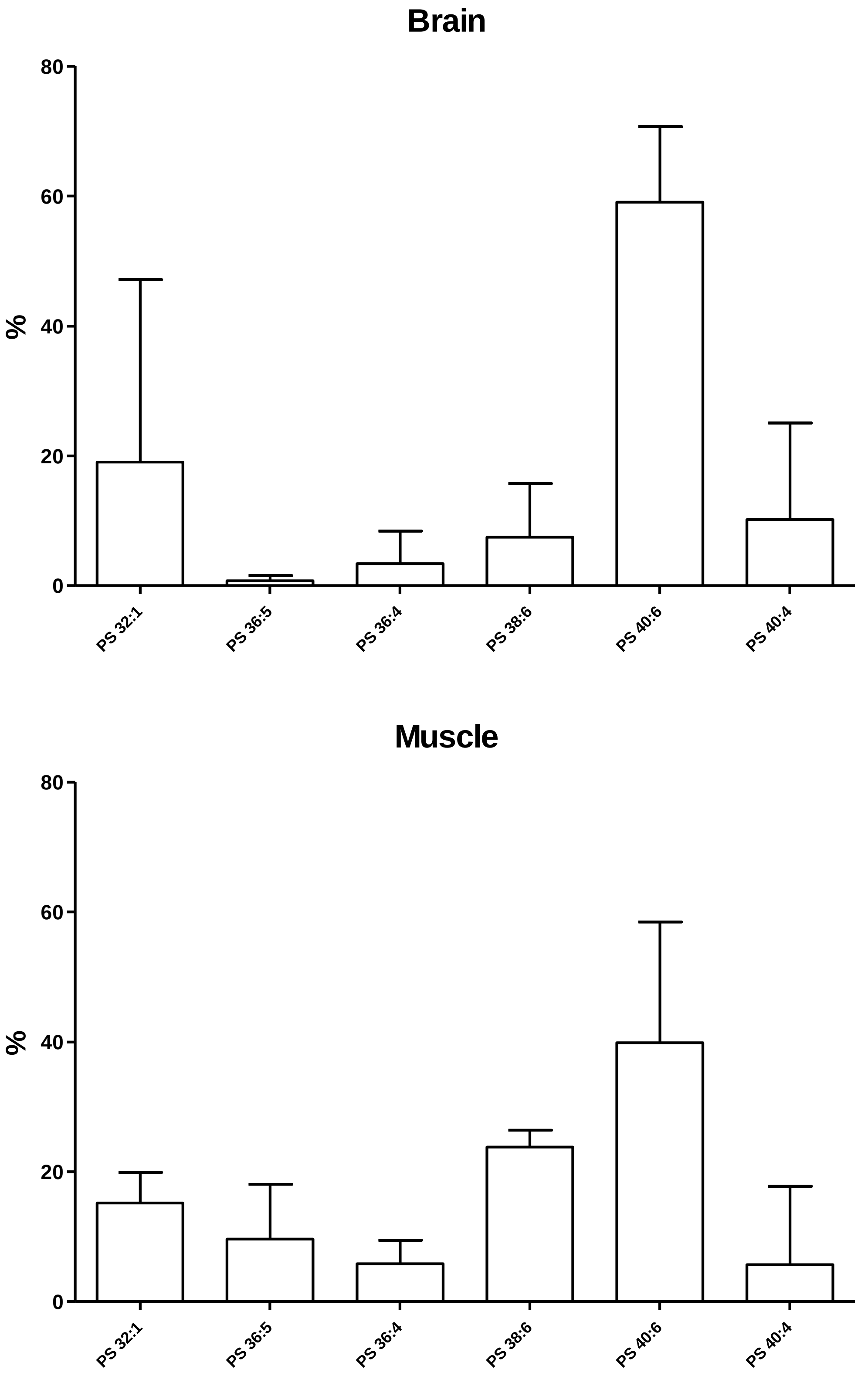

Supplementary Fig. 5

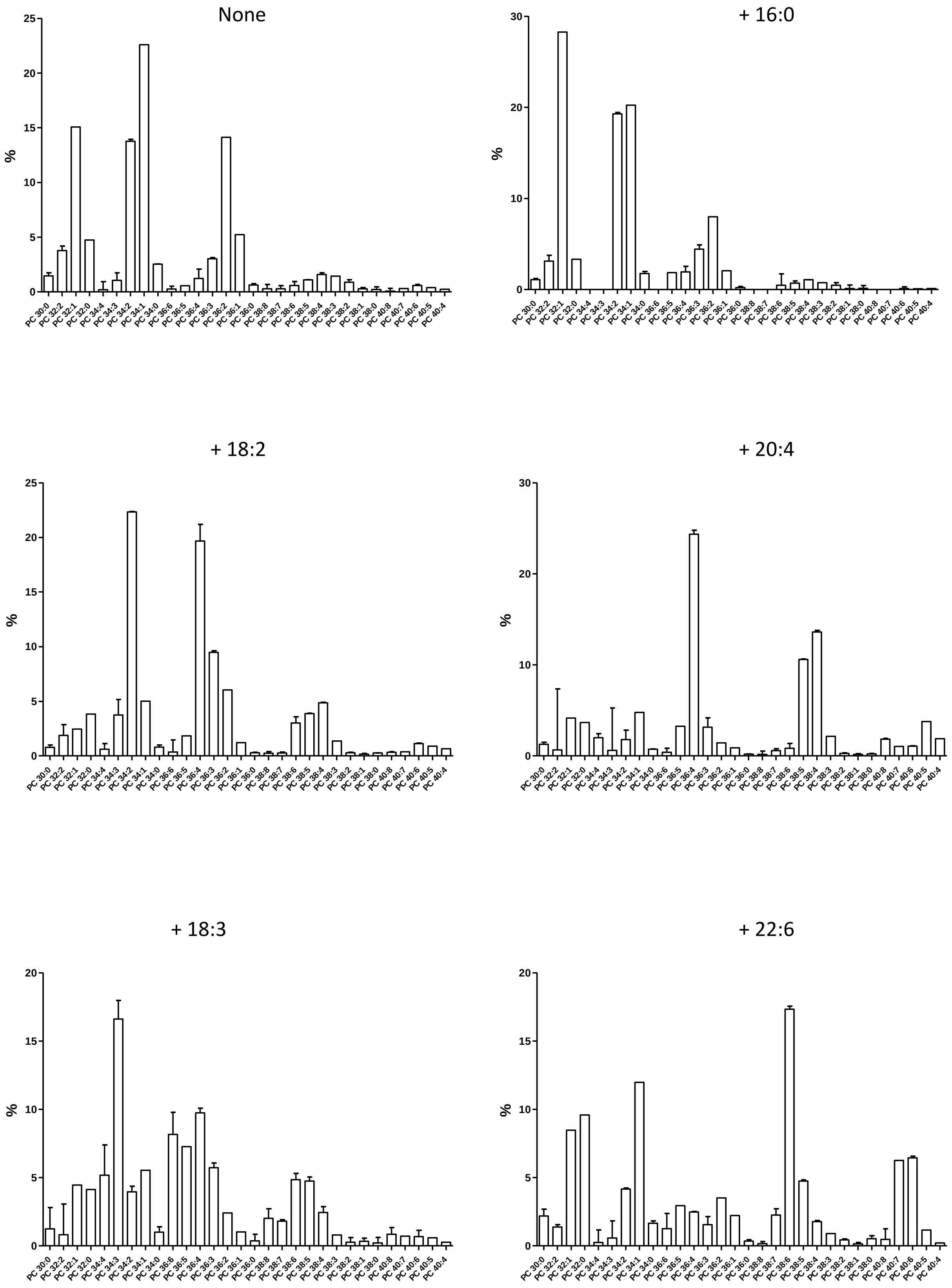

Supplementary Figure 6

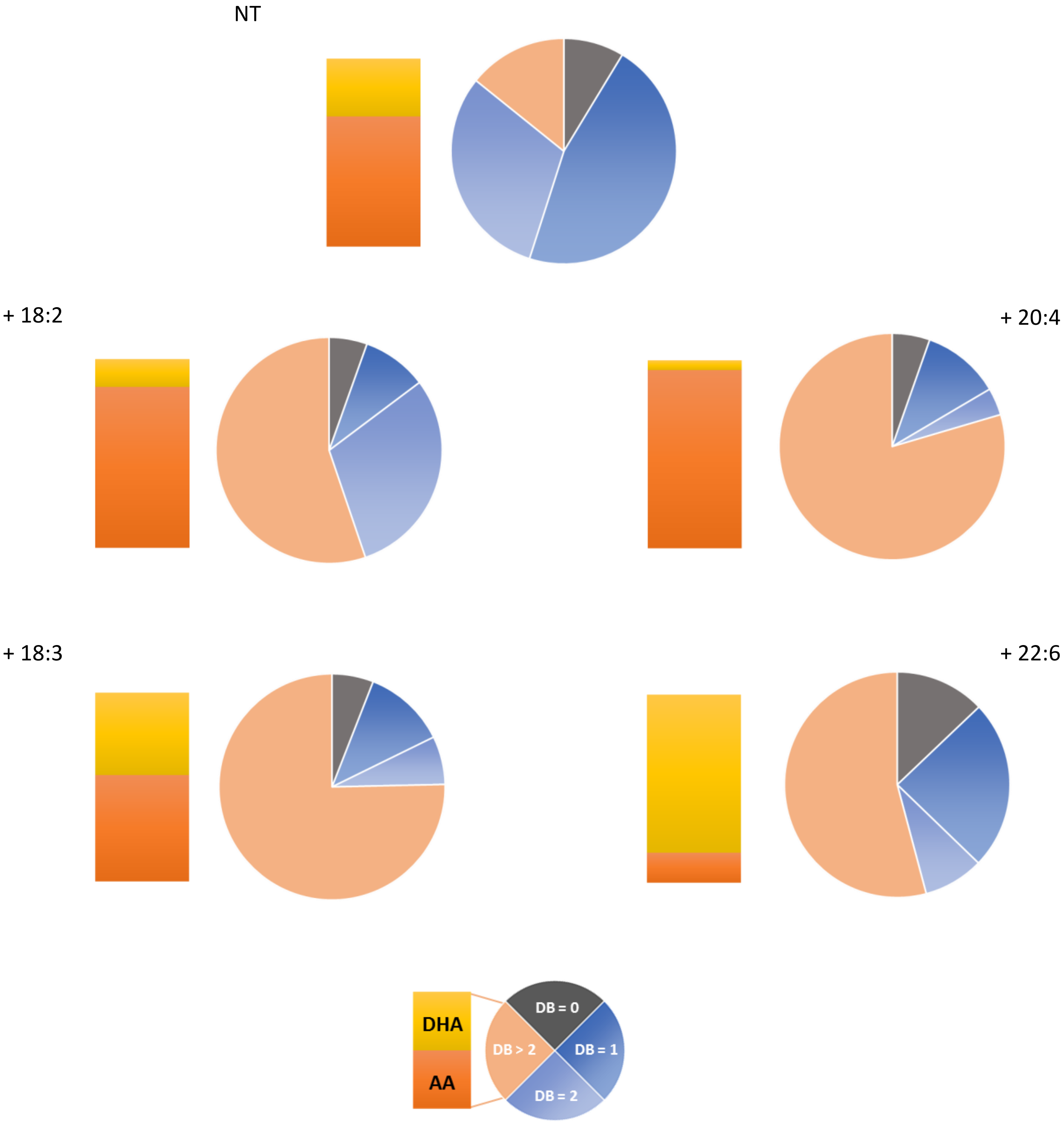

Supplement: Supplementary file 1 [file cells-10-00937-s001.zip › Supplementary Figures and Legends.pdf]
